# Two structure papers, a call from Frankfurt airport, and how to escape from reviewer delays: An interview with Peter Walter

**DOI:** 10.1186/1741-7007-11-34

**Published:** 2013-04-15

**Authors:** Peter Walter, Miranda Robertson

**Affiliations:** 1 Howard Hughes Medical Institute, University of California, San Francisco, 600 16th Street, Room S272, Box 0724, San Francisco, CA 94158, USA; 2 Department of Biochemistry and Biophysics, University of California, San Francisco, Genentech Hall, 600 16th Street, San Francisco, CA 94158, USA; 3 BMC Biology, BioMed Central, 236 Gray's Inn Road, London WC1X 8HL, UK

## 

Peter Walter is currently an Investigator of the Howard Hughes Medical Institute and a Professor in the Department of Biochemistry and Biophysics at the University of California, San Francisco (UCSF). Having earned a bachelor's degree in chemistry from the Free University of Berlin, an MS degree in organic chemistry from Vanderbilt University, he studied for his PhD in biochemistry at the Rockefeller University where he worked with Günter Blobel and identified the signal recognition particle that is required for the translocation of membrane and secreted proteins in the endoplasmic reticulum. Research in his laboratory at UCSF is now focused on protein sorting and targeting to the ER and on understanding the interplay between ER homeostasis and disease. He is a coauthor of the Molecular Biology of the Cell, published by Garland Science and now in its fifth edition. For two of those editions he worked closely with Miranda Robertson, then associated with Garland publishing and now the Editor of *BMC Biology*, to whose Editorial Board she recruited him.

**Figure:**
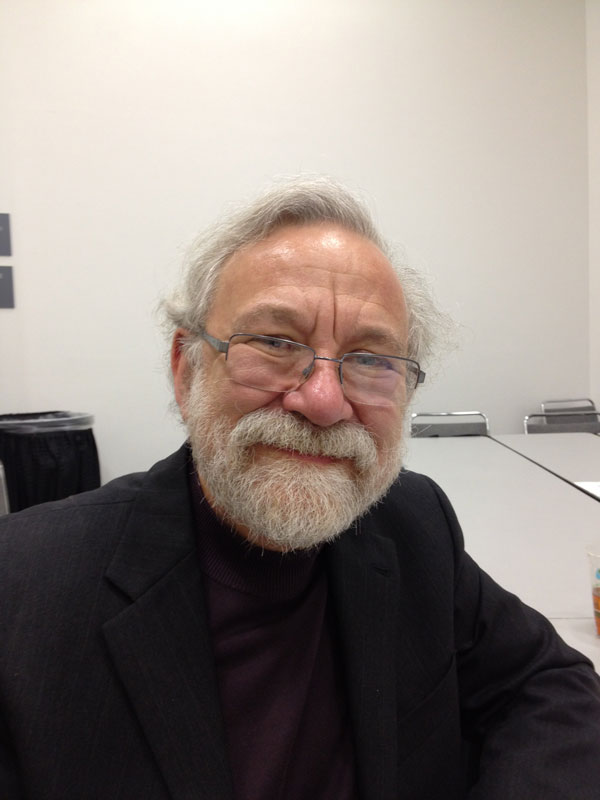
**Peter Walter**.

To mark the tenth anniversary of *BMC Biology*, she asked him to cast his mind back to a telephone call.

## MR: Peter - The seed of our re-review opt-out policy was first sown when you called me from Frankfurt airport with a rather surprising proposition: would you like to remind me what that was?

PW: Well, if I remember correctly I asked you if *BMC Biology *would publish a paper that had been submitted to and reviewed by another journal.

## MR: Oh it was worse than that - you wanted our undertaking that we would publish the paper on the basis of the existing referees' reports and without further review, because you needed that assurance before you withdrew the paper from the journal where it was still under consideration

PW: Yes.

## MR: What drove you to make that suggestion?

PW: Well I probably was completely jetlagged having arrived from the West Coast in Frankfurt and had many hours sitting there uncomfortably contemplating our frustration with this submission. The paper had been submitted, we got reviews back, we had responded to the reviewers' comments, and then we were put in limbo, it seems now for months. The paper was in re-review and the reviewer just never responded.

## MR: Before we go on with the story, would you just briefly explain what the paper was about?

PW: It was the structure of our favorite enzyme IRE1, which is a signal transmission molecule that senses unfolded proteins in the ER, and then signals in a fascinating way - it's a kinase and nuclease in the same polypeptide chain, and the structure showed how it was activated by a process of oligomerization. We had pressed the journal because it was a structure paper, and one of the reviewers requested the coordinates so he or she could look at them and then was sitting on the paper, and the science was getting stale.

## MR: Presumably also the reviewers could have used those coordinates to do their own experiments and get ahead of you

PW: Precisely. The never-ending delay was just completely unacceptable.

## MR: So to get back to the story, I asked you to give me I think a few hours to think about your proposition, that we should take these papers essentially on the basis of anonymous referees' reports, and agree to publish them, if you pulled them from the journal that they were currently with. I thought it over, discussed it with a colleague, and then emailed you "Yes we can"

## So now perhaps you'd like to say what happened to the papers in the end

PW: So basically you were showing us that we had another way we could publish this work without incurring further delays, and it gave us the strength to then send an e-mail to the journal just saying we would like to hear a decision on this paper in the next 24 hours, otherwise please consider the paper formally withdrawn. I didn't feel very good about having to put so much pressure on them, making such a strong statement.

## MR: It must also have been a risk for the postdoc whose paper it was?

PW: Yes, I incurred quite a large phone bill in discussing our options and philosophy with him, but the postdoc was a hundred percent in agreement that we should take strong and proactive steps in this case. It was a review process completely gone awry.

## MR: And what happened when you told the journal that you were going to pull the paper?

PW: In 24 hours it was accepted.

## MR: And that was a happy ending for you and your postdoc - and one way to escape from reviewing delays, though not one I think either of us would advocate in ordinary circumstances - and of course we lost the papers

PW: But you got a wonderful new reviewing policy out of it.

## MR: We did. Clearly there was a need to escape from reviewing delays, and during the few hours I asked for to think over your proposition, I confronted the question - if there is a strong case for doing this for these papers, shouldn't we be doing it for everyone's? And before I told you yes, we had resolved on a policy of allowing authors to decide whether referees should see their papers again after revision - subject to the support of our Editorial Board, who turned out to be overwhelmingly in favor

## But I do think this case - and indeed our re-review opt-out policy - raises the question, do you not think that by insisting that authors meet the requirements of referees, the journal is simply protecting the integrity of scientific reporting, and ensuring that what comes out in the journal is sound?

PW: Yes of course. But some comments are useful and make the paper better - they're actually quite rare in my experience - and other comments are just "more could be done, more could be done". Such requests put the students and postdocs into endless holding loops, where for months, or sometimes even more, they are performing reviewer experiments, in many cases orthogonal to where scientific judgment might say the research should be going, or where we would like it to go.

## MR: So your argument is that the paper should be judged on the basis of what's in that paper and not what might be in another paper if you did some more experiments

PW: Yes. I think it's one of the maladies of our current reviewing practices that reviews get longer and longer, papers get longer and longer, a lot of material is buried in the mausoleum of supplemental information that nobody reads anyway and that's not properly reviewed either, and often the main message is weakened or even lost.

What is missing is decisive editors at the other end of the process, because some reviewers' comments are quite useful, while others are simply saying what more can be done, without necessarily improving the paper. There's always more that can be done for any paper, but the research packaged in the manuscript has already been judged to be appropriate for the journal when it's sent out for review. It's the function of the reviewers to figure out if the points that we are trying to conclude are properly supported by the data that we show. It's not the function of the reviewer to say whether this paper is appropriate for the journal, that's the editor's role. I think in many cases --when things get dragged out forever and when people get asked to do endless more experiments--it's where the editor needs to step in and clearly state that while this would be a nice experiment, it will not be required for acceptance of this work. Unfortunately, in many instances we find that the editor on the other end of the line just isn't decisive enough.

## Note

This article is part of the *BMC Biology *tenth anniversary series. Other articles in this series can be found at http://www.biomedcentral.com/bmcbiol/series/tenthanniversary.

